# Ultrastructural evidence of microglial heterogeneity in Alzheimer’s disease amyloid pathology

**DOI:** 10.1186/s12974-019-1473-9

**Published:** 2019-04-16

**Authors:** Hassan El Hajj, Julie C. Savage, Kanchan Bisht, Martin Parent, Luc Vallières, Serge Rivest, Marie-Ève Tremblay

**Affiliations:** 1 Axe neurosciences, Centre de recherche du CHU de Québec-Université Laval, 2705, boulevard Laurier, T2-50, Quebec, QC G1V 4G2 Canada; 2 Département de psychiatrie et de neurosciences, Faculté de médecine, Université Laval, Quebec, QC Canada; 3 CERVO Brain Research Center, Quebec, QC Canada; 4 Département de médecine moléculaire, Faculté de médecine, Université Laval, Quebec, QC Canada

**Keywords:** Microglia, Alzheimer’s disease, Mouse model, Ultrastructure, Electron microscopy

## Abstract

**Background:**

Alzheimer’s disease (AD) is the most common neurodegenerative disease, characterized by the deposition of extracellular fibrillar amyloid β (fΑβ) and the intracellular accumulation of neurofibrillary tangles. As AD progresses, Aβ drives a robust and prolonged inflammatory response via its recognition by microglia, the brain’s immune cells. Microglial reactivity to fAβ plaques may impair their normal surveillance duties, facilitating synaptic loss and neuronal death, as well as cognitive decline in AD.

**Methods:**

In the current study, we performed correlative light, transmission, and scanning electron microscopy to provide insights into microglial structural and functional heterogeneity. We analyzed microglial cell bodies and processes in areas containing fAβ plaques and neuronal dystrophy, dystrophy only, or appearing healthy, among the hippocampus CA1 of 14-month-old APP^Swe^-PS1Δe9 mice versus wild-type littermates.

**Results:**

Our quantitative analysis revealed that microglial cell bodies in the AD model mice were larger and displayed ultrastructural signs of cellular stress, especially nearby plaques. Microglial cell bodies and processes were overall less phagocytic in AD model mice. However, they contained increased fibrillar materials and non-empty inclusions proximal to plaques. Microglial cell bodies and processes in AD model mice also displayed reduced association with extracellular space pockets that contained debris. In addition, microglial processes in healthy subregions of AD model mice encircled synaptic elements more often compared with plaque-associated processes. These observations in mice were qualitatively replicated in post-mortem hippocampal samples from two patients with AD (Braak stage 5).

**Conclusion:**

Together, our findings identify at the ultrastructural level distinct microglial transformations common to mouse and human in association with amyloid pathology.

## Introduction

Alzheimer’s disease (AD) is the most common neurodegenerative disease, affecting 47 million people in a demographically aging world [1]. The disease causes deficits in memory, learning, and thinking abilities [2]. The best neuropathological correlates of cognitive decline in AD are neuronal dystrophy and synaptic loss, which are associated with increased network excitability, notably of the hippocampus, and precede neuronal death [3, 4]. While the hippocampus is pivotal for long-term memory formation in addition to its essential role in memory storage and retrieval [5], hippocampal atrophy is consistent in AD with a mean volume loss ranging from 20 to 50% [6]. The main pathological hallmarks of AD are the deposition of extracellular amyloid beta (Aβ) plaques, comprising insoluble Aβ fibrils (fAβ), and intraneuronal fibrillar tangles of tau protein [7]. The soluble amyloid beta (sAβ) forms a halo around fAβ plaques and is linked to neuronal dystrophy and synaptic loss, both in AD human samples and mouse models of Aβ deposition [8–13].

Genome-wide association studies (GWAS) have uncovered a strong genetic involvement of the immune system in AD, including functional variants of triggering receptor expressed on myeloid cells 2 (TREM2) [14–16]. Microglia, the resident innate immune cells of the brain, recognize and respond to both sAβ and fAβ using complexes of pattern recognition receptors [17]. Downstream signaling induces tumor necrosis factor alpha (TNFα) and interleukin-1 beta (IL-1β) secretion, which recruits nearby microglial cells via chemotaxis and can initiate a runaway inflammatory response that injures neurons as well as triggers synaptic loss [18–21]. In the healthy brain, the complement cascade mediates the pruning of less active synapses, while in aging and AD, this pathway takes a pathological role and promotes synaptic loss [20]. Microglia are firmly connected to brain inflammation and they likely contribute to its harmful long-term consequences leading to neurodegeneration and cognitive decline [9]. Microglia are also instrumental in slowing down the plaque growth and reducing synaptic loss by preventing Aβ diffusion and toxicity [22, 23], while they perform valuable roles in the clearance of both sAβ and fAβ [24, 25]. Microglial contribution to Aβ clearance can prevent the precipitation of Aβ into fibrillar plaques and reduce the brain concentration of harmful sAβ species responsible for neurodegenerative effects; a number of therapeutic studies have thus focused on increasing microglial capacity to clear sAβ and fAβ [26–28]. Additionally, microglia were involved in extracellular degradation of Aβ both in vitro and in vivo [29, 30].

Microglial implication in the pathological process of AD is contextual [31, 32] and heterogeneous, as illustrated by recent single cell transcriptomic studies [33]. For instance, the disease-associated microglia (DAM) and microglia neurodegenerative phenotype (MGnD) transcription signatures contain reduced levels of homeostatic genes, higher levels of phagocytic genes, and were shown to associate with fAβ plaques in AD mouse models and human samples [34–36]. They are also regulated by TREM2 [37]. The functional outcomes of these altered microglial transcriptomes largely remain undetermined, however, and this leaves open the question of whether and how these phenotypes clear Aβ and apoptotic neurons. Using ultrastructural analysis, our group recently described the dark microglia [36], which extensively contact synapses and are characterized by their electron-dense cytoplasm and nucleoplasm in electron microscopy. These cells are rare in the mature brain during normal physiological conditions, but they become abundant in aging and in the APP^Swe^-PS1Δe9 mouse model of amyloid deposition. In this AD model, dark microglia often associated with plaques, expressed TREM2, contained fibrillar materials, and encircled dystrophic neurites and synaptic elements. Interestingly, intermediate stages between the dark and typical microglia were observed, while the typical microglia displayed several ultrastructural alterations in APP^Swe^-PS1Δe9 mice.

To provide further insights into the implication of microglial diversity in AD, the present study aimed to define the spatial heterogeneity of microglial ultrastructure with relation to fAβ plaques, as well as neuronal dystrophy and synaptic loss, using APP^Swe^-PS1Δe9 mice [38]. This well-established model deposits fAβ plaques within the cortex and hippocampus beginning at 4 months of age, with cognitive deficits and synaptic loss detected at 6 months, and robust memory deficits by 12 months [37–40]. In the current study, we investigated microglial ultrastructure among subregions of the ventral hippocampus CA1 with fAβ plaques and neuronal dystrophy, neuronal dystrophy without plaques, or that appeared unaffected by Aβ pathology. We used a combination of correlative light, transmission, and scanning electron microscopy [41]. The APP^Swe^-PS1Δe9 mice were compared to wild-type littermate controls at 14 months of age, when sufficient plaque deposition allowed to study all three subregions simultaneously, within the same tissue sections. We focused on the CA1 *strata radiatum* and *lacunosum*-*moleculare*, two layers containing the apical dendrites of CA1 pyramidal cells, where the dark microglia were previously shown to be abundant [36]. In addition, microglial ultrastructure was analyzed in the post-mortem hippocampus of two patients with AD (Braak stage 5). Our observations reveal various microglial changes in response to amyloid pathology, some of which being specific to areas containing fAβ plaques (e.g., endosomes enclosing elements), to areas showing neuronal dystrophy without fAβ plaques (e.g., association with extracellular debris), or to areas appearing devoid of pathology (increased contacts with synaptic elements). Together, our overall findings suggest that while microglia undergo global ultrastructural changes over the course of amyloid pathology, the local environment largely determines their diversity.

## Methods

### Animals

All experimental procedures were performed in agreement with the guidelines of the Institutional Animal Ethics committees, in conformity with the Canadian Council on Animal Care and the Animal Care Committee of *Université Laval*. The animals were housed under a 12-h light-dark cycle at 22–25 °C with free access to food and water. Fourteen-month-old APP^Swe^-PS1Δe9 mice on a C57Bl/6J background (*N* = 4) were compared with age-matched wild-type littermate controls (*N* = 3). The transgenic mice coexpress human presenilin one variant (A246E) and a chimeric mouse/human amyloid precursor protein (APPSwe) [37]. Only males were used in this study considering sex differences in amyloid deposition that were described in APP^Swe^-PS1Δe9 mice [42].

APP^Swe^-PS1Δe9 mice were injected with Methoxy-X04 (10 g/kg; Tocris Bioscience) 24 h prior to sacrifice as previously described [41]. Methoxy-X04 is a Congo Red fluorescent derivative that binds to β sheets with high affinity. Mice were anesthetized with sodium pentobarbital (80 mg/kg, i.p.) and transcardially perfused with ice-cold phosphate-buffered saline (PBS; 50 mM at pH 7.4) followed by 3.5% acrolein and 4% paraformaldehyde both diluted in phosphate buffer (PB; 100 mM at pH 7.4). Transverse sections of the brain (50-μm-thick) were cut in PBS using a vibratome (Leica VT1000S) and kept in a cryoprotectant solution containing glycerol and ethylene glycol at − 20 °C until further processing [41, 43].

### Screening brain sections for fAβ plaques using light microscopy

With the aid of a stereotaxic mouse brain atlas [44], brain sections containing the ventral hippocampus CA1 (Bregma − 2.75 to − 3.5) were selected and placed into a 24-well plate. Each section was assigned an individual well. Using an ultraviolet filter with a range of 380–480 nm, an inverted Nikon Eclipse TE300 microscope was used to visualize the methoxy-X04 stained plaques at a magnification of × 4. Pictures of the sections were captured both in bright and fluorescent field modes, naming the images according to the sections position into the 24-well plate [41]. Brain sections from the age-matched controls containing the same level of ventral hippocampus as the Methoxy-X04-labeled sections were selected for comparison.

### Human tissue

Hippocampal sections containing the CA1 from two human cases of AD (C-0032, 76 years of age, 24-h post-mortem delay; H-17, 81 years of age, 6-h post-mortem delay) were obtained from the brain bank established at the CERVO Brain Research Center. The two samples are considered typical cases of AD with similar levels of pathology, staged 5 out of 6 on the Braak scale. In the two samples, Bielschowsky staining revealed moderate numbers of fAβ plaques, especially in the parietal and temporal lobes. With regard to the hippocampus, an overall atrophy was noted, together with a moderate number of fAβ plaques. Brain banking and post-mortem tissue handling procedures were approved by the *Ethic Committee of the Institut Universitaire en santé mentale de Québec* and by *Université Laval*. Brains were obtained with written consent and the analyses performed in conformity with the Code of Ethics of the World Medical Association (Declaration of Helsinki). Brains were first cut in half along the midline and hemibrains were sliced into 2-cm-thick slabs along the coronal plane. These slabs were fixed by immersion in 4% paraformaldehyde at 4 °C for 3 days. They were stored at 4 °C in PBS (100 mM at pH 7.4) containing 15% sucrose and 0.1% sodium azide. Slabs containing the hippocampus CA1 were cut with a freezing microtome into 50-μm-thick sections that were serially collected in PBS and stored at − 20 °C in a cryoprotectant solution containing glycerol and ethylene glycol until use.

### Brain sections immunostaining and processing for electron microscopy

Hippocampal sections from the human cases, wild-type littermate controls, and APP^Swe^-PS1Δe9 mice containing Aβ plaques in the CA1 were selected for immunohistochemical (immuno)EM staining. They were washed in PBS, quenched with 0.3% hydrogen peroxide (H_2_O_2_) in PBS, and washed again. Sections were then incubated with 0.1% solution of NaBH_4_ for 30 min at room temperature (RT). After several washes, sections were incubated in a blocking buffer of Tris-buffered saline (TBS; 50 mM at pH 8.0) containing 10% fetal bovine serum, 3% bovine serum albumin, and 0.01% Triton X100 for 2 h at RT. Sections were incubated overnight at 4 °C in rabbit anti-ionized calcium binding adaptor molecule 1 (IBA1) antibody (1:1000 in blocking solution; Wako Pure Chemical Industries) and rinsed in TBS. The sections were afterwards incubated for 1.5 h in goat anti-rabbit IgGs conjugated to biotin (1:200 in TBS; Jackson Immunoresearch) and 1 h in the ABC Vectastain system kit (1:100 in TBS; Vector Laboratories) for immunoperoxidase staining. The staining was revealed using diaminobenzidine (DAB; 0.05%) and H_2_O_2_ (0.015%) in TBS for 8 min. Sections were post-fixed flat in 1% osmium tetroxide in PB for 30 min and dehydrated in increasing concentrations of ethanol, immersed in propylene oxide, impregnated in Durcupan resin (Electron Microscopy Sciences; EMS) overnight at RT, and polymerized between ACLAR films (EMS) at 55 °C for 72 h. Areas of interest were excised from the embedded sections, glued to resin blocks [41], and sectioned at 70–80 nm using a Leica UC7 ultramicrotome.

### Transmission electron microscopy imaging

Ultrathin sections collected on square mesh grids (EMS) were imaged using a FEI Tecnai Spirit G2 microscope equipped with an ORCA-HR Hamamatsu (10MP) camera. Microglial cell bodies were identified using a series of distinctive features that comprise their electron density, association with extracellular space pockets, characteristic long stretches of endoplasmic reticulum (ER), numerous vacuoles and intracellular inclusions, irregular contours with obtuse angles, and small elongated nucleus delineated by narrow nuclear cisternae [45, 46]. In most cases, they were also identified by their IBA1 immunoreactivity. Our analysis did not distinguish between the yolk-sac derived microglia and circulating myeloid cells that infiltrate the brain [47].

For qualitative analysis, pictures were acquired at magnifications ranging from × 1400 to × 13,000. Both mouse and human hippocampal samples were analyzed qualitatively to investigate microglial interactions with the synaptic neuropil, including contacts with dystrophic neurites and dendritic spines, phagocytosis, and association with extracellular space pockets containing debris. For quantitative analysis in the mouse samples, pictures of microglial cell bodies and processes were randomly acquired, at × 6800 and × 9300, respectively. In the APP^Swe^-PS1Δe9 mice, three different subregions were differentiated. The first subregion contained one fAβ plaque, with some rare cases showing two fAβ plaques, accompanied by neuronal dystrophy within a 113 μm × 113 μm grid square; the second subregion displayed neuronal dystrophy notably observed as clusters of swelled neurites containing autophagic vacuoles as well as electron dense bodies but no fAβ plaque within a 113 μm × 113 μm grid square; and the third subregion only comprised ultrastructurally unaffected neuronal and glial cell compartments with intact plasma membranes and organelles, comparable to the wild-type control samples, within a 113 μm × 113 μm grid square. In the third subregion, myelinated axons were also surrounded by compact sheaths of myelin without ultrastructural alteration. While great care was taken in defining these three subregions in our samples, and the plaques were identified in light microscopy prior to ultrathin sectioning, it is possible that neuronal dystrophy was present outside of the imaged section. The same number of microglial cell bodies and processes was imaged in the wild-type controls, ranging from 7–15 microglial cell bodies and 50–70 microglial processes per subregion/animal. To ensure that some of the larger microglial processes were not, in fact, proximal processes containing part of the soma, a 2 μm^2^ area was instituted as an upper bound for microglial process size.

### Scanning electron microscopy array tomography imaging

In order to image microglia near fAβ plaques in the human tissue, scanning electron microscopy (SEM) with array tomography was used. The same tissue was used for both transmission electron microscopy (TEM) and SEM imaging. However, after ultrathin sections were made, they were collected onto polished silicon chips and treated with 2% osmium tetroxide in 1.5% potassium ferrocyanide solution, followed by 1% thiocarbohydrazide, and 1% osmium, as described in the National Center for Microscopy and Imaging Research serial blockface SEM protocol [48]. Following this post-fixation, the chips were mounted onto pin stubs using double-sided conductive carbon tape and imaged in a Zeiss Crossbeam 540 SEM, using acceleration voltage of 1.4 kV and current of 1.2 nA, at a maximum magnification of 5 nm per pixel.

### Quantitative ultrastructural analysis of microglia

The analysis of microglial cell bodies and processes comprised several ultrastructural measures of morphology, phagocytic activity, cellular stress, and physiological function. Experimenters were blinded to the experimental conditions throughout the analysis. Size and shape descriptors were determined using ImageJ. For each microglial cell body and process, phagocytic inclusions (appearing electron-lucent and named “empty” versus containing materials and named “non-empty”), lysosomes (primary, secondary, versus tertiary), lipid bodies, fibrillar materials, ER dilation, vacuoles (diameter < 100 nm), and extracellular space pockets containing debris were counted, according to the quantitative code 0, 1, 2, and 3+ (designating 3 and more occurrences). Lysosomes were identified by their dense, heterogeneous contents enclosed by a single membrane [49]. Primary lysosomes possessed a homogenous granular content and their diameter ranged from 0.3 to 0.5 μm [50]. Secondary lysosomes were 1 to 2 μm across, and their content was heterogeneous showing fusion with vacuoles. Tertiary lysosomes ranged in diameter between 1.5 and 2.5 μm, and they were usually fused to one or two vacuoles associated with lipofuscin granules, as well as lipidic inclusions showing signs of degradation [51]. Lipidic inclusions were identified as the clustering of round organelles with an electron dense, either opaque or limpid, cytoplasm enclosed by a single membrane. Lipofuscin granules were identified by their oval or round shapes, finely granular composition, and associated amorphous materials [51]. Extracellular fAβ was identified as densely packed fibrils and filaments, according to previous ultrastructural descriptions [52]. ER dilation was recognized by a swelling of the cisternal space ranging from 50 to 300 nm [53]. Extracellular space pockets containing debris, which could result from “exophagy” (degradation of cellular constituents by lysosomal enzymes released extracellularly), exocytosis (the process of expelling the contents of a membrane-bound vesicle into the extracellular space, often lysosomal and in preparation for phagocytosis; [54]), or pinocytosis (also named bulk-phase endocytosis, by which cells can take up extracellular contents in a non-phagocytic manner; [55]) was defined by the appearance of degraded materials (including cellular membranes and organelles) or debris in the extracellular space juxtaposing the microglia [54].

For microglial processes, their encirclement of neuropil compartments (axon terminals, dendritic spines, synapses between axon terminals and dendritic spines, cellular elements with signs of degradation) was also quantified. Encirclement was defined as microglial interactions with these neuropil compartments that displayed at least two points of contact, sometimes extending over several hundreds of nanometers. They were scored using the quantitative code 0, 1, 2, and 3+ (designating 3 and more elements). The encircled elements were identified according to the following criteria: axon terminals contained synaptic vesicles and were frequently seen branching from axons or making synapses onto dendritic branches and spines, and dendritic spines were identified as extensions from dendrites often forming synapses where a postsynaptic density was observed. Moreover, microglial encirclement of extracellular debris was determined.

### Statistical analysis

GraphPad Prism 7 was used for statistical analysis. The APP^Swe^-PS1Δe9 mice were compared to age-matched wild-type littermate controls using a non-parametric Mann-Whitney test. Microglial cell bodies and processes were further compared between plaque-associated, neuronal dystrophy associated, and ultrastructurally healthy tissue of APP^Swe^-PS1Δe9 mice using a non-parametric Kruskal-Wallis test without matching, followed by Dunn’s multiple comparisons post-hoc test. Data are expressed as mean ± standard error of the mean (SEM) [56, 57]. The sample size (*n*) refers to individual microglial cell bodies or processes as previously performed for quantitative ultrastructural analyses [56, 58–60]. Statistically significant differences are indicated by *,~,#*p* < 0.05, **,~~,##*p* < 0.01, and ***,~~~,###*p* < 0.001.

## Results

### Microglial ultrastructure is altered in proximity to fAβ

**Fig. 1:**
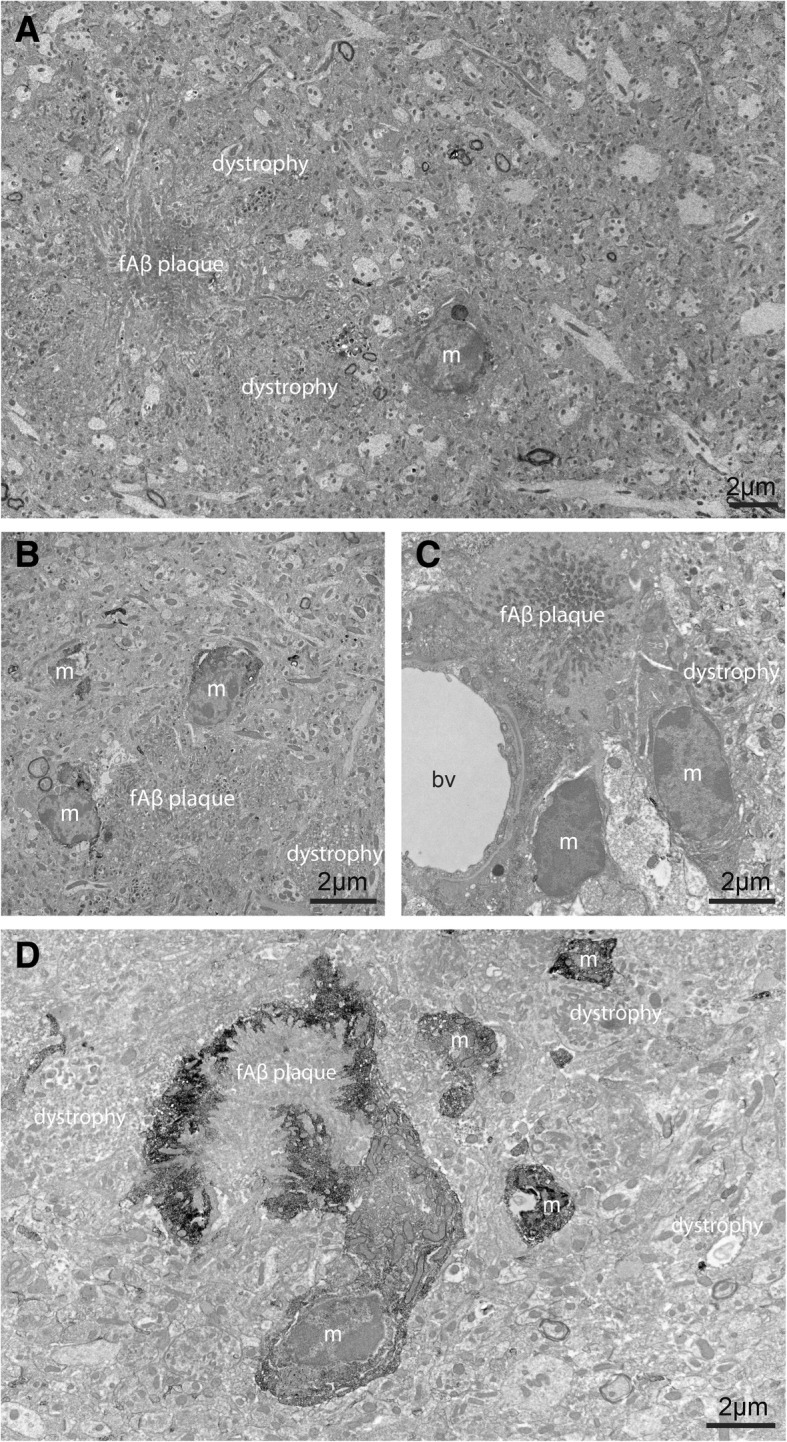
Defining dystrophic and plaque-associated neuropil areas. **a** An example EM image displaying a microglial cell body in plaque- and dystrophic-associated region of the ventral hippocampus CA1 in APP^Swe^-PS1Δe9 model mice. **b** Two microglial cell bodies and a microglial process showing IBA1 immunoreactivity surround a fAβ plaque and associated dystrophy. **c** A microglial cell body contacts both a dystrophic neurite and a fAβ plaque while a second microglial cell contacts a blood vessel. **d** A microglial cell body and its proximal process displaying IBA1 immunoreactivity encircle a fAβ plaque. Other microglial processes, discontinuous to the cell body in ultrathin section, can also be seen. *bv* blood vessel, *m* microglia. Dystrophy and fAβ plaques are labeled throughout the figure

**Fig. 2:**
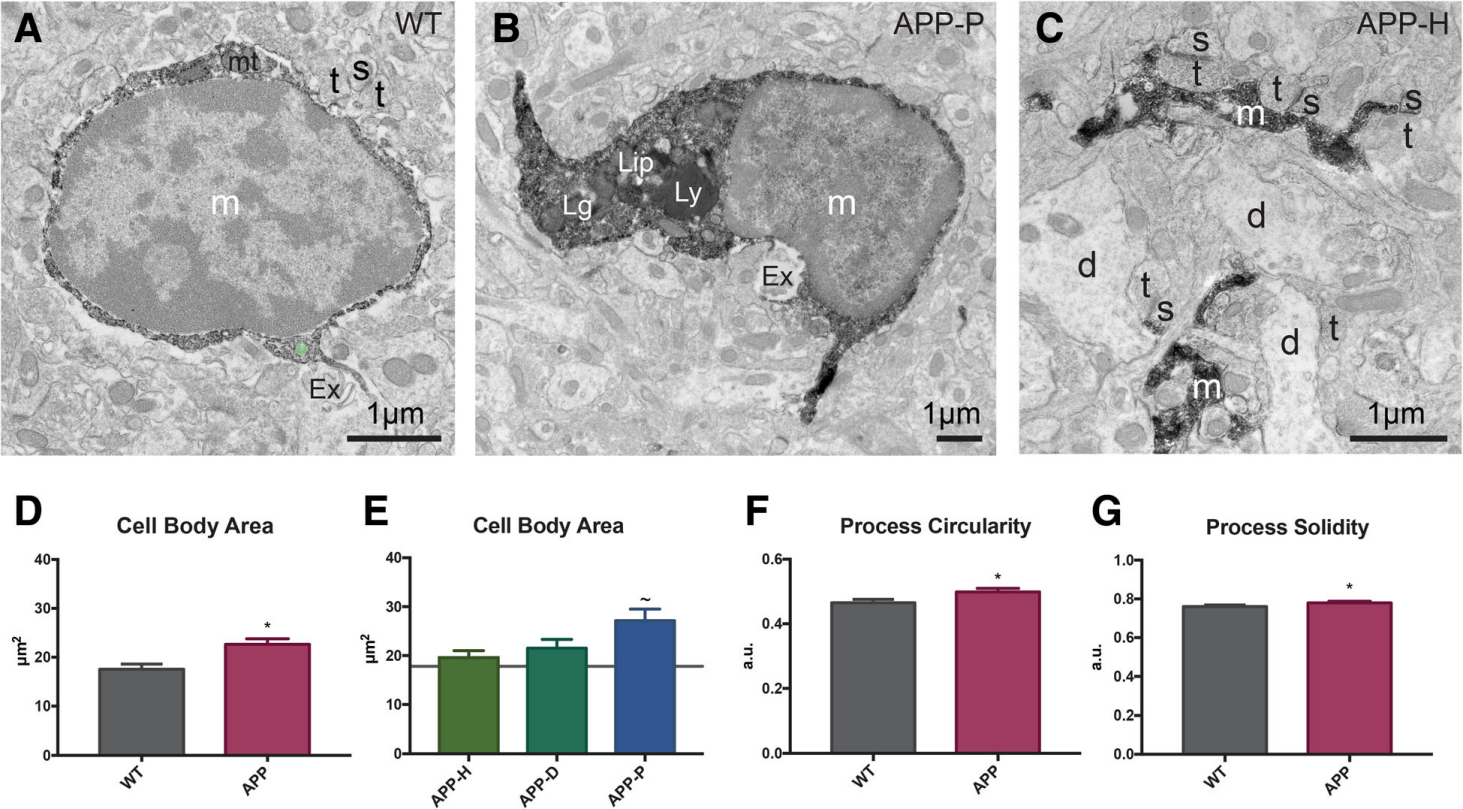
Microglial ultrastructure is changed by proximity to fAβ plaques. **a** EM image from a WT control animal showing oval shaped IBA1-positive microglial cell body contacting extracellular space pocket and axon terminal in the ventral hippocampus CA1. **b** EM image of a plaque-associated microglial cell body that contains lipidic inclusions, lipofuscin granule, and lysosome, as well as contacting extracellular space pocket. **c** Microglial processes in an ultrastructurally healthy subregion of APP^Swe^-PS1Δe9 mouse model, touching or encircling various types of synaptic elements. *d* dendrite, *Ex* extracellular space pocket containing debris, *Lg* lipofuscin granule, *Lip* lipidic inclusion, *Ly* lysosome, *m* microglia, *mt* mitochondrion, *s* dendritic spine, *t* axon terminal. **d** Change in microglial cell body area of WT controls compared to APP^Swe^-PS1Δe9 animals. **e** Change in microglial cell body area in APP-H, APP-D, and APP-P subregions. **f** Change in the microglial process circularity in WT and APP^Swe^-PS1Δe9 animals. **g** Change in the microglial process solidity in WT and APP^Swe^-PS1Δe9 animals. *n* = 50 processes per condition for APP-H, APP-D, and APP-P and 70 processes per animal for WT animals, *n* = 7–15 cells per condition for all conditions, and data was collected from *N* = 3–4 animals per condition. All error bars are mean ± SEM. WT levels are shown on the subregion graphs by a gray horizontal line. *p < 0.05 difference from WT, ~*p* < 0.05 difference from APP-H

**Fig. 3:**
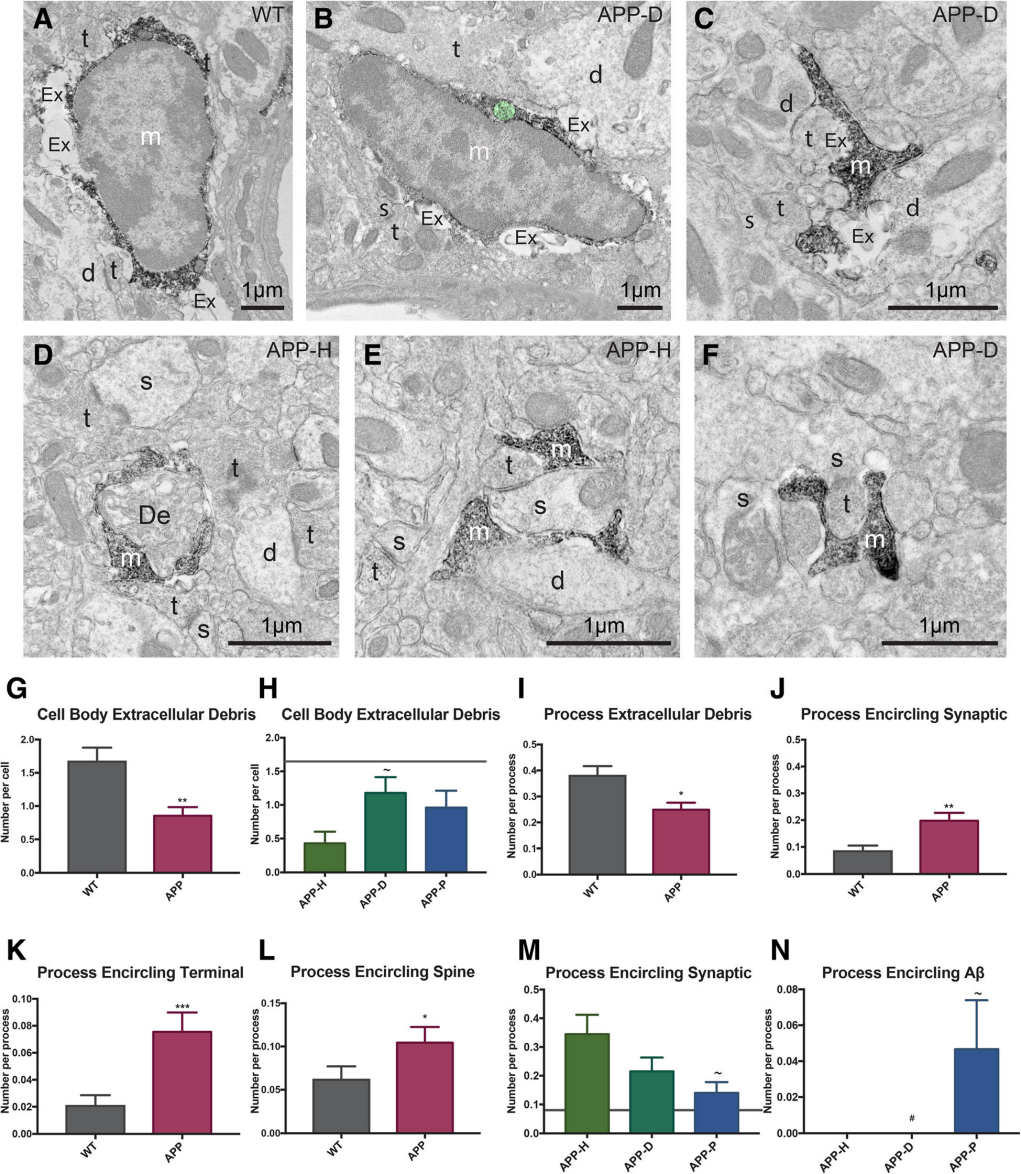
Distinct alterations in microglial interaction with the neuropil, including synaptic structures, in APP^Swe^-PS1Δe9 animals. **a** EM image of an IBA1-positive microglial cell body in the ventral hippocampus CA1 of a WT control making numerous contacts with extracellular space pockets containing debris. **b** EM image of an IBA1-positive microglial cell body in a section containing dystrophy, making contact with extracellular space pockets and an axon terminal. A non-empty phagocytic inclusion is pseudocolored in green. **c** An IBA1-positive microglial process makes contacts with extracellular space pockets containing debris, as well as various dendrites and axon terminals. **d** IBA1-positive microglial process encircling degraded elements in the APP-H subregion. **e**, **f** IBA1-positive microglial processes encircling synapses between axon terminals and dendritic spines in APP-H and APP-D subregions. *d* dendrite, *De* degraded element, *Ex* extracellular space pocket containing debris, *m* microglia, *s* dendritic spine, *t* axon terminal. **g** Microglial cell bodies interact less with extracellular debris in APP^Swe^-PS1Δe9 mice. **h** Microglial cell bodies in APP-H regions interact less with extracellular debris than those in APP-D regions in APP^Swe^-PS1Δe9 mice. **i** Microglial processes in APP^Swe^-PS1Δe9 mice interact less with extracellular debris than WT processes. **j**–**l** Processes in APP^Swe^-PS1Δe9 mice encircle more synaptic elements (**j**) including (**k**) axon terminals, and (**l**) dendritic spines. **m** Processes in APP-P associated regions encircle fewer synaptic elements than processes in APP-H subregion. **n** Only microglial processes in APP-P subregion encircle Aβ (mean ± SEM). WT levels are shown on the subregion graphs by a gray horizontal line. *n* = 50 processes per condition for APP-H, APP-D, and APP-P and 70 processes per animal for WT animals, *n* = 7–15 cells per condition for all conditions, and data was collected from *N* = 3–4 animals per condition. * denotes difference from WT, ~ denotes difference from APP-H, # denotes difference from APP-P *,~,#*p* < 0.05; **p < 0.01; ***p < 0.001

**Fig. 4:**
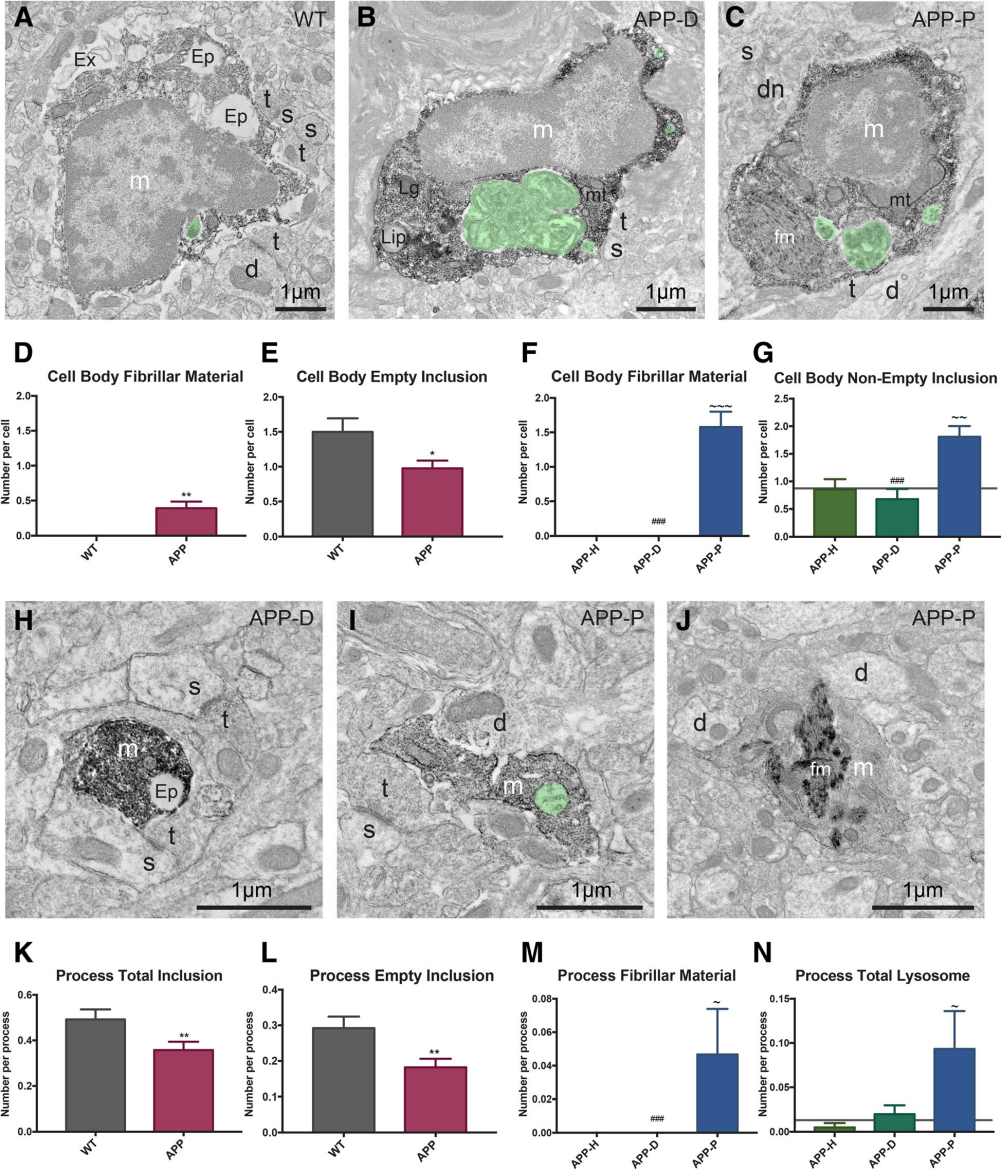
Distinct changes in microglial phagolysosomal system of APP^Swe^-PS1Δe9 animals. **a**–**c** Example of IBA1-positive microglial cell bodies containing empty inclusions, non-empty inclusions (pseudocolored in green), or fibrillar materials in wild-type (WT; **a**), APP-D (**b**), or APP-P (**c**) subregions of ventral hippocampus CA1. **d** Quantification of fibrillar materials phagocytosis by microglial cell bodies in APP^Swe^-PS1Δe9 mice. **e** Quantification of cell bodies containing empty phagocytic inclusions in APP^Swe^-PS1Δe9 mice. **f** Only microglial cell bodies in APP-P regions contained fibrillar materials. **g** Cell bodies in APP-P regions contained increased numbers of non-empty phagocytic inclusions. **h**–**j** IBA1-positive microglial processes containing various phagocytic inclusions in APP-P and APP-D subregions. **k** Quantification of microglial process inclusions in WT and APP^Swe^-PS1Δe9 mice. **l** Quantification of microglial processes containing empty inclusions in APP^Swe^-PS1Δe9 mice. **m** Only microglial processes in APP-P regions contained fibrillar materials. **n** Quantification of lysosome numbers in APP-H, APP-D, and APP-P subregions associated microglial processes. *d* dendrite, *Ep* empty phagocytic inclusion, *dn* dystrophic neurite, *fm* fibrillar material, *Ex* extracellular space pocket containing debris, *m* microglia, *mt* mitochondrion, *s* dendritic spine, *t* axon terminal. Error bars represent mean ± SEM. WT levels are shown on the subregion graphs by a gray horizontal line. *n* = 50 processes per condition for APP-H, APP-D, and APP-P and 70 processes per animal for WT animals, *n* = 7–15 cells per condition for all conditions, and data was collected from *N* = 3–4 animals per condition. * denotes difference from WT, ~ denotes difference from APP-H, # denotes difference from APP-P *,~*p* < 0.05; **,~~*p* < 0.01; ***,~~~,###*p* < 0.001

**Fig. 5:**
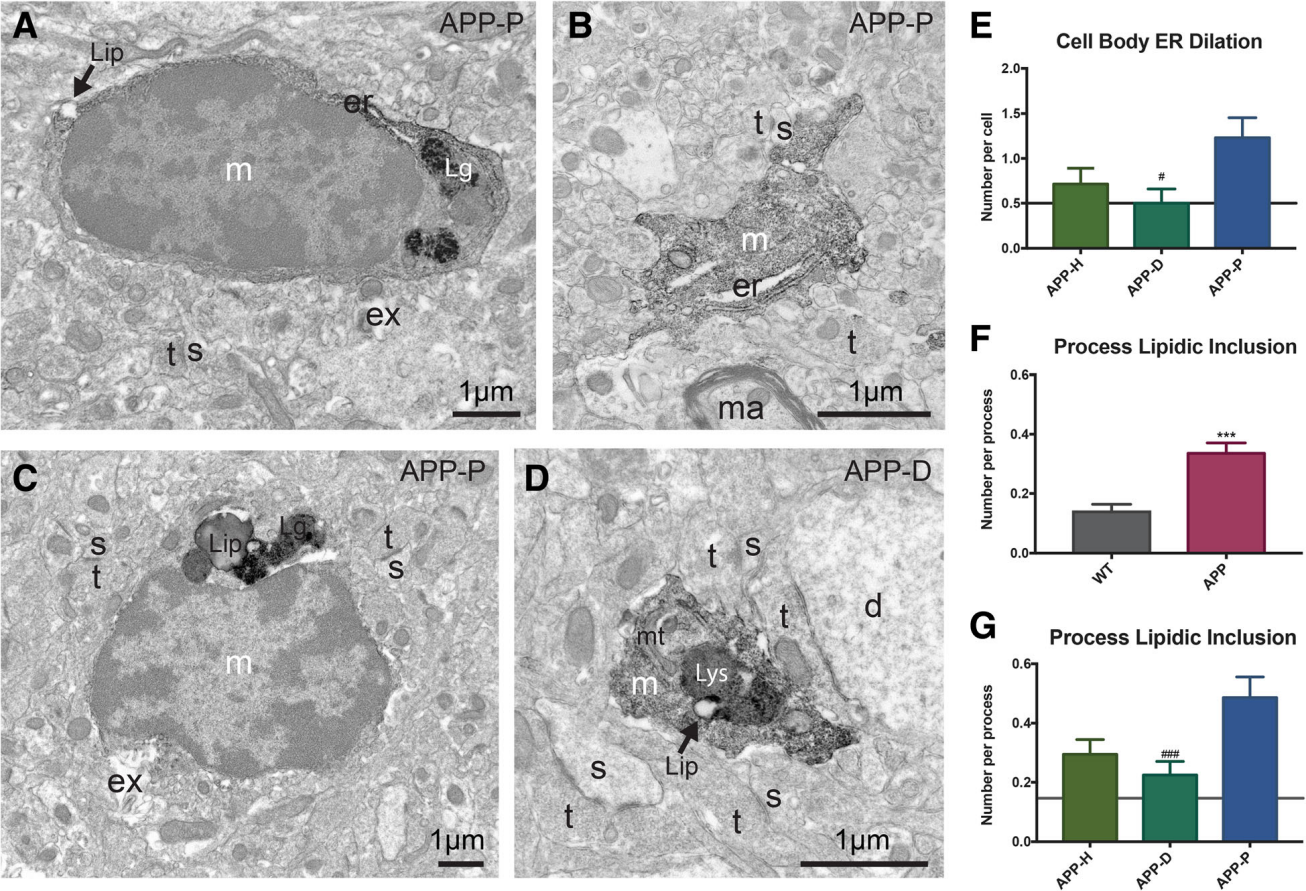
Microglia in APP^Swe^-PS1Δe9 animals display increased signs of cellular stress. **a** Example of IBA1-positive cell body imaged in the ventral hippocampus CA1 of the APP-P subregion that displays ER dilation, a lipidic inclusion and lipofuscin granules. **b** Example of microglial process in the APP-P subregion showing ER dilation. **c** IBA1-positive microglial cell body contacting extracellular space pockets with debris and containing lipidic inclusions and lipofuscin granules. **d** Microglial cell process containing a lysosome and a lipidic inclusion. *d* dendrite, *er* endoplasmic reticulum, *Ex* extracellular space pocket containing debris, *Lg* lipofuscin granule, *Lip* lipidic inclusion, *Lys* lysosome, *m* microglia, *ma* myelinated axon, *mt* mitochondrion, *s* dendritic spine, *t* axon terminal. **e** Quantification of dilated ER in microglial cell bodies from APP-H, APP-D, and APP-P subregions of APP^Swe^-PS1Δe9 mice. **f** Microglial processes in APP^Swe^-PS1Δe9 animals are more likely to contain lipidic inclusions than those in WT animals. **g** Quantification of lipid body inclusions in microglial processes from APP-H, APP-D, and APP-P subregions. *n* = 50 processes per condition for APP-H, APP-D, and APP-P and 70 processes per animal for WT animals, *n* = 7–15 cells per condition for all conditions, and data was collected from *N* = 3–4 animals per condition. Error bars represent mean ± SEM. WT levels are shown on the subregion graphs by a gray horizontal line. #*p* < 0.05; ***,###*p* < 0.001. * denotes difference from WT animal, # denotes difference from APP-P region

We characterized at nano-scale resolution microglial ultrastructure among subregions containing fAβ plaques, displaying neuronal dystrophy, or appearing healthy, among the hippocampus CA1 *strata radiatum* and *lacunosum*-*moleculare* of 14-month-old APP^Swe^-PS1Δe9 mice versus wild-type littermates (Fig. 1). Microglial cell bodies displayed, in all four conditions, characteristic bean-shaped nuclei containing patches of dense heterochromatin, while their cytoplasm showed variable levels of immunoEM reactivity against the marker IBA1 (Fig. 1a–d). In line with previous descriptions of microglial ultrastructure [46], their cell bodies contained numerous mitochondria often in close apposition to characteristically long stretches of ER, together with occasional Golgi apparati and phagocytic vesicles (Figs. 1d, 2a–c, 3a, b, 4a–c, and 5a, c). When studying whole-region changes in microglial ultrastructure, cell body area was significantly different between wild-type and transgenic mice, showing increase in APP^Swe^-PS1Δe9 animals (Fig. 2d, Table 1). Similar findings were obtained by separating the APP^Swe^-PS1Δe9 investigation into subregions (ultrastructurally healthy, dystrophic, and plaque-associated with dystrophy) (Fig. 2e, Table 2).

**Table 1:** Ultrastructural analysis of microglial cell bodies in wild-type (WT) littermate controls versus APP^Swe^-PS1Δe9 mice

	WT	APP
	Mean ± SEM	Mean ± SEM
Area (μm^2^)	17.53 ± 1.06	22.64 ± 1.13*
Perimeter (μm)	21.98 ± 1.01	25.71 ± 1.41
Circularity (a.u.)	0.482 ± 0.02	0.487 ± 0.018
Roundness (a.u.)	0.594 ± 0.027	0.606 ± 0.187
Solidity (a.u.)	0.838 ± 0.0139	0.838 ± 0.01
Lipid body (*n*)	0.667 ± 0.159	0.488 ± 0.10
Fibrillar materials inclusion (*n*)	0	0.390 ± 0.096**
Phagocytic (%)	78.6 ± 6.40	79.3 ± 4.50
Total inclusion (*n*)	1.810 ± 0.187	1.707 ± 0.123
Non-empty inclusion (*n*)	0.833 ± 0.148	1.024 ± 0.119
Empty inclusion (*n*)	1.50 ± 0.194	0.976 ± 0.111
ER dilation (*n*)	0.50 ± 0.11	0.80 ± 0.11
Extracellular debris (*n*)	1.667 ± 0.212	0.854 ± 0.131**
Primary lysosome (*n*)	0.1667 ± 0.108	0.171 ± 0.064
Secondary lysosome (*n*)	0.762 ± 0.186	0.548 ± 0.109
Tertiary lysosome (*n*)	0.643 ± 0.17	0.402 ± 0.096
Total lysosome (*n*)	1.00 ± 0.21	0.89 ± 0.138

*a*.*u*. arbitrary unit, *n* number per microglial cell body

**Table 2:** Ultrastructural analysis of microglial cell bodies in healthy (APP-H), dystrophic (APP-D), and plaque-associated with dystrophy (APP-P) regions of APP^Swe^-PS1Δe9 mice

	APP-H	APP-D	APP-P
	Mean ± SEM	Mean ± SEM	Mean ± SEM
Area (μm^2^)	19.58 ± 1.53	21.52 ± 1.80	27.15 ± 2.38~
Perimeter (μm)	23.08 ± 1.24	23.87 ± 1.64	30.5 ± 3.76
Circularity (a.u.)	0.482 ± 0.026	0.519 ± 0.033	0.459 ± 0.034
Roundness (a.u.)	0.546 ± 0.03	0.647 ± 0.032	0.625 ± 0.031
Solidity (a.u.)	0.836 ± 0.018	0.856 ± 0.018	0.822 ± 0.194
Lipid body (*n*)	0.75 ± 0.22	0.429 ± 0.158	0.269 ± 0.142
Fibrillar materials inclusion (*n*)	0	0	1.577 ± 0.223~~~###
Phagocytic (%)	0.75 ± 0.083	0.786 ± 0.079	0.846 ± 0.072
Total inclusion (*n*)	1.536 ± 0.209	1.536 ± 0.215	2.077 ± 0.228
Non-empty inclusion (*n*)	0.857 ± 0.18	0.679 ± 0.186	1.808 ± 0.192~~###
Empty inclusion (*n*)	0.857 ± 0.191	1.071 ± 0.205	1.00 ± 0.184
ER dilation (*n*)	0.714 ± 0.177	0.500 ± 0.159	1.231 ± 0.224#
Extracellular debris (*n*)	0.429 ± 0.174	1.179 ± 0.236~	0.962 ± 0.251
Primary lysosome (*n*)	0.286 ± 0.162	0.174 ± 0.050	0.154 ± 0.091
Secondary lysosome (*n*)	0.679 ± 0.21	0.50 ± 0.174	0.462 ± 0.177
Tertiary lysosome (*n*)	0.75 ± 0.213	0.321 ± 0.146	0.115 ± 0.085~
Total lysosome (*n*)	1.25 ± 0.27	0.714 ± 0.22	0.577 ± 0.185

*a*.*u*. arbitrary unit, *n* number per microglial cell body. ~ significant difference from APP-H; # significant difference from APP-D

Microglial cell bodies of wild-type and APP^Swe^-PS1Δe9 mice were significantly different in size, but their morphology remained largely unchanged (Table 3). Numerous in vivo two-photon imaging studies revealed that microglial processes are incredibly dynamic as they survey the brain neuropil via continuous extension and retraction [61]. Microglial processes are long, thin, often ramified, discontinuous from cell bodies in ultrathin sections, and are identified by their immunoEM staining for IBA1, which diffuses throughout their cytosol (Figs. 2c, 3c–e, 4h–j, and 5b, d). They often accumulate phagocytic vesicles, and more proximal processes also contain mitochondria, occasional ER and/or Golgi apparati, as well as lysosomes. While the area of microglial processes did not differ between genotypes (Table 3), processes from APP^Swe^-PS1Δe9 mice had significantly rounder and more solid shape descriptors (Fig. 2f, g and Table 3).

**Table 3:** Ultrastructural analysis of microglial processes in wild-type (WT) littermate controls versus APP^Swe^-PS1Δe9 mice

	WT	APP
	Mean ± SEM	Mean ± SEM
Area (μm^2^)	0.4111 ± 0.021	0.3957 ± 0.020
Perimeter (μm)	3.476 ± 0.13	3.409 ± 0.13
Circularity (a.u.)	0.465 ± 0.1	0.499 ± 0.01*
Roundness (a.u.)	0.483 ± 0.009	0.508 ± 0.009
Solidity (a.u.)	0.761 ± 0.008	0.780 ± 0.007*
Lipidic inclusion (*n*)	0.139 ± 0.025	0.336 ± 0.035***
Fibrillar materials inclusion (*n*)	0	0.0133 ± 0.007
Phagocytic process (%)	0.3179 ± 0.024	0.2289 ± 0.02
Total inclusion (*n*)	0.4923 ± 0.043	0.3578 ± 0.037
Non-empty inclusion (*n*)	0.210 ± 0.026	0.189 ± 0.027
Empty inclusion (*n*)	0.292 ± 0.032	0.182 ± 0.024
ER dilation (*n*)	0.021 ± 0.008	0.04 ± 0.009
Extracellular debris (*n*)	0.380 ± 0.038	0.249 ± 0.027*
Total lysosome (*n*)	0.0153 ± 0.0062	0.0422 ± 0.015
Primary lysosome (*n*)	0.005 ± 0.003	0.009 ± 0.007
Secondary lysosome (*n*)	0.01 ± 0.005	0.03 ± 0.009
Tertiary lysosome (*n*)	0	0.016 ± 0.008
Encircled Aβ (*n*)	0	0.016 ± 0.009
Encircled synaptic element (*n*)	0.085 ± 0.02	0.20 ± 0.03**
Encircled dendritic spine (*n*)	0.06 ± 0.016	0.10 ± 0.018*
Encircled axonal terminal (*n*)	0.02 ± 0.008	0.08 ± 0.01***
Encircled degraded structure (*n*)	0.015 ± 0.007	0.029 ± 0.009

*a*.*u*. arbitrary unit, *n* number per microglial process

Although their gross ultrastructural features were not significantly different between wild-type and APP^Swe^-PS1Δe9 mice, microglial cell bodies and processes interacted differently with their surrounding neuropil depending on the genotype. Strikingly, microglial cell bodies and processes in APP^Swe^-PS1Δe9 mice reduced their association with extracellular space pockets containing debris (Fig. 3a, b, g, i). This debris contained cellular membranes that sometimes formed vesicles (see Figs. 3b, c and 4a). In the healthy adolescent brain, pockets of extracellular space are tightly associated with microglial cell bodies and processes, without noticeable accumulation of materials [45]. Whether this accumulation of debris observed here is associated with exophagy, exocytosis, or macropinocytosis events [54, 55, 62] warrants further investigation. In addition, differences were observed based on microglial cell body location among subregions of fAβ plaques, neuronal dystrophy, or apparent health (Fig. 3h). Microglial processes in APP^Swe^-PS1Δe9 mice similarly reduced their association with extracellular debris (Fig. 3c, i, Table 3). Microglial processes in APP^Swe^-PS1Δe9 animals nevertheless encircled more dendritic spines and axon terminals than processes in wild-type animals (Fig. 3e, f, j–m). This increase in encirclement was also noted in conjunction with degraded structures (Fig. 3d), though the trend did not reach statistical significance (Table 3). However, in the subregions of fAβ plaques, microglial processes were less likely to encircle synaptic elements, while more likely to encircle fibrils of amyloid (Fig. 3m, n). This data indicates that microglia respond to global changes (i.e., increased interactions with synapses in AD model), but still halt normal surveillance duties when in the immediate presence of fAβ.

### Microglial phagocytosis is reduced in AD model mice

One of microglias' most pivotal roles in the development and maintenance of the brain is their capacity to recognize and actively phagocytose, and degrade extraneous synapses and apoptotic cells. Microglia often contain phagocytic inclusions in their cell bodies and processes. Using immunoEM, the cargos contained within the phagocytic vesicles of microglia can be identified (Fig. 4a–c). Microglial cell bodies in APP^Swe^-PS1Δe9 contained fibrillar materials (Fig. 4c, d), only when they were located nearby plaques (Fig. 4f). Empty inclusions appear as electron lucent, while non-empty inclusions are electron dense, and sometimes can be recognized as surrounding membranes, vesicles, or myelin sheaths (Fig. 4a–c). Microglial cell bodies in APP^Swe^-PS1Δe9 mice contained significantly fewer empty inclusions than wild-type animals (Fig. 4e). In contrast, microglial cell bodies located near fAβ plaques were more likely to contain non-empty inclusions as compared with ultrastructurally healthy subregions (Fig. 4g).

Microglial phagocytosis begins with a microglial membrane wrapping and engulfing its phagocytic target. The vesicle is then trafficked to the soma via the phagolysosomal pathway, undergoing degradation along the way. As expected, the ratio of phagosomes containing non-empty to empty inclusions was higher within microglial processes than microglial somas (Tables 1, 2, 3, and 4). Upon investigation, we found that microglial processes in APP^Swe^-PS1Δe9 mice were not less phagocytic than the processes from wild-type animals (Table 3). However, their total number of inclusions (both empty and non-empty) were significantly reduced in numbers in APP^Swe^-PS1Δe9 mice (Fig. 4k, l). Comparing subregions, fibrillar materials were observed only in processes near plaques (Fig. 4m).

**Table 4:** Ultrastructural analysis of microglial processes in healthy (APP-H), dystrophic (APP-D), and plaque and dystrophy (APP-P) containing regions of APP^Swe^-PS1Δe9 mice

	APP-H	APP-D	APP-P
	Mean ± SEM	Mean ± SEM	Mean ± SEM
Area (μm^2^)	0.361 ± 0.026	0.451 ± 0.033	0.485 ± 0.037
Perimeter (μm)	3.333 ± 0.197	3.575 ± 0.2	3.317 ± 0.209
Circularity (a.u.)	0.486 ± 0.016	0.501 ± 0.017	0.488 ± 0.018
Roundness (a.u.)	0.485 ± 0.014	0.527 ± 0.014	0.492 ± 0.016
Solidity (a.u.)	0.768 ± 0.012	0.789 ± 0.012	0.775 ± 0.125
Lipidic inclusion (*n*)	0.295 ± 0.050	0.225 ± 0.046	0.487 ± 0.07##
Fibrillar materials inclusion (*n*)	0	0	0.047 ± 0.027~#
Phagocytic process (%)	0.2 ± 0.028	0.255 ± 0.031	0.24 ± 0.035
Total inclusion (*n*)	0.295 ± 0.046	0.4 ± 0.056	0.38 ± 0.687
Non-empty inclusion (*n*)	0.115 ± 0.029	0.19 ± 0.038	0.193 ± 0.041
Empty inclusion (*n*)	0.18 ± 0.035	0.21 ± 0.04	0.147 ± 0.038
ER dilation (*n*)	0.025 ± 0.011	0.05 ± 0.154	0.033 ± 0.0175
Extracellular debris (*n*)	0.24 ± 0.04	0.255 ± 0.039	0.24 ± 0.047
Total lysosome (*n*)	0.005 ± 0.005	0.002 ± 0.01	0.0933 ± 0.04~
Primary lysosome (*n*)	0	0.02 ± 0.016	0
Secondary lysosome (*n*)	0.005 ± 0.005	0.01 ± 0.007	0.06 ± 0.027~
Tertiary lysosome (*n*)	0	0.01 ± 0.007	0.033 ± 0.024
Encircled Aβ (*n*)	0	0	0.047 ± 0.027~#
Encircled synaptic element (*n*)	0.345 ± 0.067	0.215 ± 0.049	0.14 ± 0.038~
Encircled dendritic spine (*n*)	0.185 ± 0.038	0.115 ± 0.028	0.08 ± 0.026
Encircled axonal terminal (*n*)	0.155 ± 0.028	0.09 ± 0.024	0.053 ± 0.018
Encircled degraded structure (*n*)	0.025 ± 0.013	0.04 ± 0.017	0.006 ± 0.006

*a*.*u*. arbitrary unit, *n* number per microglial process. ~ significant difference from APP-H; # significant difference from APP-D

To provide additional insights into the alteration of microglial degradation activity, we next investigated the prevalence and determined the stage of lysosomes in microglial cell bodies and processes (Tables 1, 2, 3, and 4). While primary lysosomes are not commonly found in microglial somas (0.167 per cell body, Table 1), secondary and tertiary lysosomes containing phagocytic or degraded materials were often present in both wild-type and APP^Swe^-PS1Δe9 animals (between 0.40 and 0.76 per cell body, Table 1). Interestingly, total lysosomes as well as specifically secondary lysosomes increased in microglial processes from plaque-associated areas (Figs. 4n, 5d, Table 4). Although the number of fibrillar materials inclusions was elevated in the APP^Swe^-PS1Δe9 animals (Table 1), the numbers of primary, secondary, and tertiary lysosomes per cell body and process were not significantly different with respect to amyloid pathology (Tables 1 and 2).

### Effects of Aβ on microglial cell stress

The best characterized ultrastructural marker of cellular stress is ER stress, read out at high spatial resolution as a lumen dilation of ER and Golgi apparatus cisternae. It has been described in several neurodegenerative diseases, including amyotrophic lateral sclerosis and AD [63, 64]. ER dilation is also characteristic of the dark microglia, a phenotype upregulated in disease contexts including AD pathology [36]. In the current study, we were interested in investigating intermediate stages between the typical microglia and dark ones. Various hallmarks of dark microglia were examined in the ventral hippocampus CA1 *strata radiatum* and *lacunosum*-*moleculare* of wild-type and APP^Swe^-PS1Δe9 animals, including the presence of dilated ER among microglial cell bodies and processes, the alteration to organelles including mitochondria, the condensation of cytoplasmic and nucleoplasmic contents (thought to result in a dark/electron-dense appearance), and the loss of heterochromatin pattern.

Healthy ER is characterized by long stretches of parallel membranes with very little space between them. On the other hand, dilated ER appears with non-parallel membranes containing bloated stretches of electron-lucent materials (Fig. 5a, b). When comparing the prevalence of dilated ER among microglial cell bodies from APP^Swe^-PS1Δe9 animals and wild-type controls, there was no difference between genotypes (Table 1). After separating our analysis into healthy, neuronal dystrophy, or fAβ plaque subregions, it became clear that an increase in dilated ER was present, but only in plaque-associated microglial cell bodies (Fig. 5e).

In addition to studying dilated ER in microglial cell bodies and processes, we investigated the presence of lipidic inclusions in microglia from WT and APP^Swe^-PS1Δe9 animals. Lipidic inclusions are dynamic clusters of lipid droplets, made up of a cholesterol and triglyceride core surrounded by a phospholipid monolayer, which house numerous signaling proteins, and are visible in electron microscopy as circular droplets of homogeneous electron density (Fig. 5a, c, d). Lipid droplets are actively formed in microglial cells that respond to pro-inflammatory cytokines and may represent a marker of cellular metabolic stress or an early hallmark of neuroinflammation [56]. The number of lipidic inclusions significantly increased in microglial processes from APP^Swe^-PS1Δe9 animals, especially nearby plaques (Fig. 5f, g).

In addition, microglial cell bodies often displayed reduced IBA1 immunoreactivity in the APP^Swe^-PS1Δe9 animals, across the three subregions (see Figs. 5a, c and 1), while the dark microglia were previously shown to downregulate IBA1 in the same hippocampal region of 14-month-old APP^Swe^-PS1Δe9 mice [36]. Microglial cell bodies endowed with dilated ER, as observed around the plaques mainly, sometimes appeared darker than the typical microglia (see Figs. 1c and 5a), suggesting slight condensation of their cytoplasmic and nucleoplasmic contents. These darker cells however had a clearly defined heterochromatin pattern, as compared with the dark microglia.

### Microglial ultrastructure in human cases of AD

**Fig. 6:**
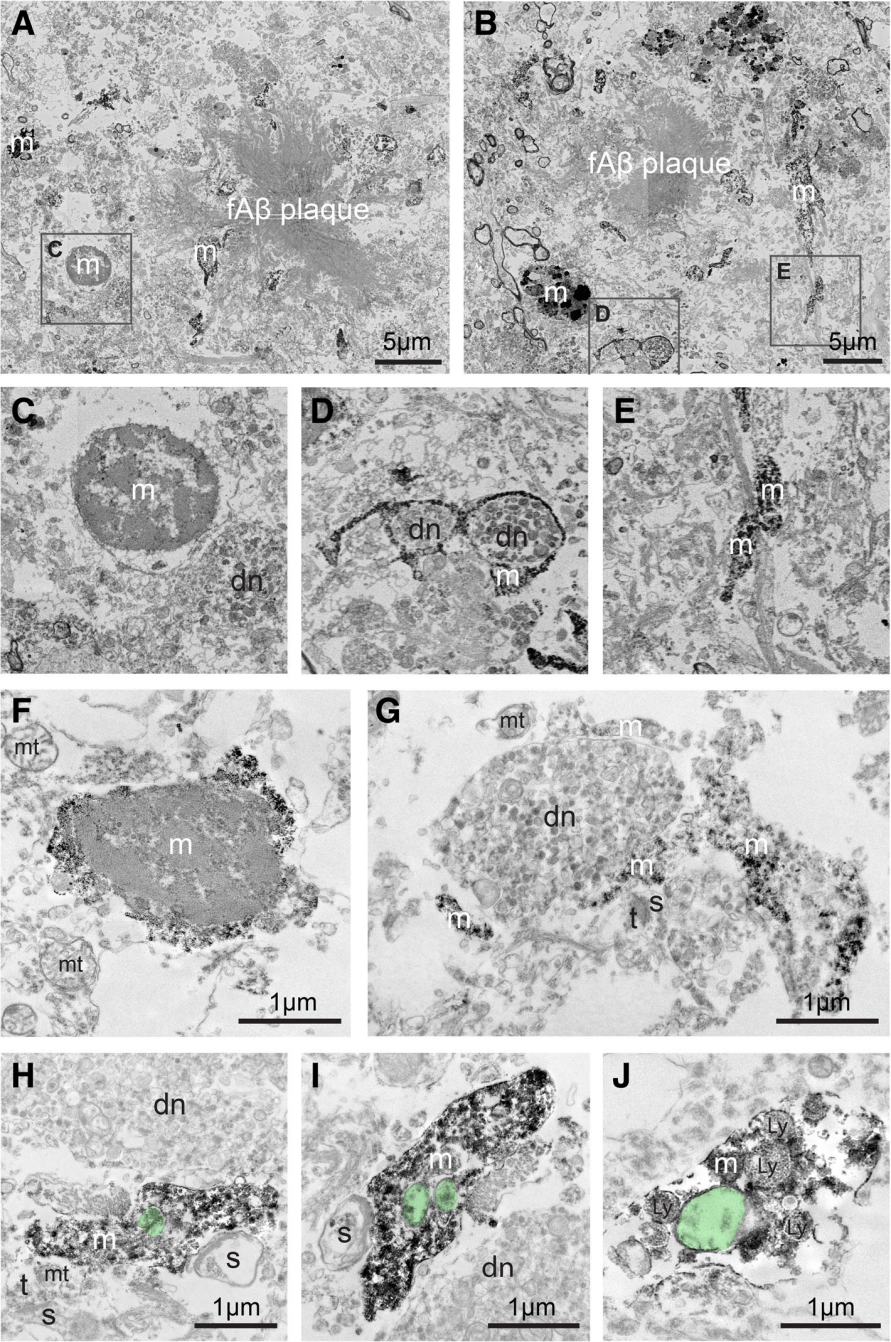
Microglial ultrastructure in *post-mortem* hippocampal samples from two human AD patients. **a**, **b** Examples of IBA1-immunopositive microglial cell bodies and processes surrounding fAβ plaques in the *post-mortem* hippocampus from an individual diagnosed with AD (Braak stage 5). **c**–**e** Insets from A and B, showing: **c** a microglial cell body contacting a dystrophic neurite, **d** a microglial process encircling two dystrophic neurites, and **e** another microglial process wrapping around other neuropil elements. **f**–**j** IBA1-immunopositive microglial cell body and processes from *post-mortem* hippocampal tissue from a second individual diagnosed with AD (also Braak stage 5). **g**–**j** Microglial processes seen making contacts with dystrophic neurites and dendritic spines, some of them receiving synapses from axon terminals, as well as containing phagocytic inclusions and lysosomes. *dn* dystrophic neurite, *Ly* lysosome, *m* microglia, *mt* mitochondrion, *s* dendritic spine, *t* axon terminal. Non-empty phagocytic inclusions are pseudocolored in green

In an effort to determine the clinical relevance of our microglial ultrastructure studies conducted in mouse hippocampus, we investigated tissue gathered from the hippocampi of two post-mortem AD cases. Fifty-micrometer-thick sections containing the hippocampus were stained against IBA1 to identify microglial cell bodies and processes. Occasional fAβ plaques were observed (Fig. 6a, b), with abundant dystrophic neurites (Fig. 6a–d, h, i), recognized by their accumulation of small, regularly packed dense bodies, and numerous autophagic vacuoles [65]. This identification of fAβ plaques and dystrophic neurites in human samples is consistent with the literature [65–68]. As such, the tissues we investigated from AD individuals closely paralleled the subregions of fAβ plaques and neuronal dystrophy that were analyzed in APP^Swe^-PS1Δe9 mice.

Microglia in human AD cases were found to be immunoreactive for IBA1 in both their processes and cell body, and they displayed the same characteristic nuclei described in mice (Fig. 6c, f). Microglial processes were often seen to contact dystrophic neurites and dendritic spines (Fig. 6d, g–j). Interestingly, microglial processes were observed making several contacts and nearly completely encircling multiple sides of a dystrophic neurite containing an accumulation of autophagic vacuoles (Fig. 6g). Microglia in human tissue from patients who suffered from AD were also phagocytic, with many processes displaying one or more phagocytic inclusions (Fig. 6h–j). These phagocytic inclusions appeared either empty or non-empty, consistent with the inclusions described in mouse microglia. Lysosomes were also frequently observed in the human microglial processes, showing intact granular structure and often juxtaposing vesicular inclusions, suggesting a probable fusion to produce a phagolysosome (Fig. 6j). In addition, human microglial cell bodies and processes frequently associated with pockets of extracellular space that contained cellular debris, at various stages of degradation, supporting microglial involvement with the removal of extracellular debris (Fig. 6c–j). Microglia in human AD cases thus recapitulate several ultrastructural characteristics seen in the mouse model, particularly their intimate interactions with fAβ plaques, dystrophic neurites, and dendritic spines.

## Discussion

Unraveling the role of microglia has been complicated by the heterogeneity displayed by these cells across brain regions, stages of the lifespan, and contexts of health and disease. The past decade has been a boon in the description of microglial phenotypic diversity and plasticity, using in vivo two-photon microscopy, single-cell RNA sequencing, and positron emission tomography to find hotspots of microglial activity within the human brain [69]. However, these techniques have been unable to investigate intimate microglial interactions with their surrounding brain environment. We have utilized TEM and SEM in order to uncover region-specific roles of microglia in the APP^Swe^-PS1Δe9 mouse model of AD pathology. Our data revealed striking ultrastructural differences of microglia among ventral hippocampus CA1 subregions containing fAβ plaques, dystrophic neurons, or appearing ultrastructurally healthy, from APP^Swe^-PS1Δe9 mice, versus wild-type littermate controls. These findings were qualitatively replicated in two individuals who suffered from AD (Braak stage 5).

By separating AD pathology into separate subregions (healthy, dystrophic, and plaque-associated), we were able to discern subtle, but statistically significant, changes of microglial cell body and process morphology. Microglial cell bodies close to fAβ plaques were larger than in any other tissue investigated. Microglial processes profiles were also rounder and increased their solidity in AD model mice, regardless of dystrophic neurons or plaques proximity. These findings are concordant with the microglial morphological changes that were described at the light level in mouse models of amyloid pathology [70], and with the two-photon in vivo imaging studies demonstrating microglia from AD mouse models have reduced process motility [71, 72].

Our analyses in healthy subregions of hippocampus devoid of fAβ plaque and dystrophic neurons revealed microglial characteristics of reduced extracellular degradation, accompanied by increased encirclement of dendritic spines. Studies in humans with mild cognitive impairment and in mouse models of AD have uncovered links between memory impairment and hyperactive neuronal networks within the hippocampus [73–75]. The loss of dendritic spines in the CA1 *strata radiatum* and *lacunosum*-*moleculare* is a leading mechanism linked to neuronal network hyperactivity in 10- to 14-month-old ARTE10 model mice [3]. Interestingly, the encirclement of dendritic spines was validated using our human AD hippocampal samples. It is thus tempting to hypothesize that microglia’ increased contacts with neurons and synapses could be exacerbating cognitive impairment. Numerous in vitro and in vivo studies across various models of AD have identified profound synaptic deficits in response to small oligomeric and sAβ [8]. Increased levels of sAβ are hypothesized to predispose neurons to both hyperactivity and excitotoxicity, which are associated with increases in local microglial response [9, 76]. Recent human studies have even implicated fAβ plaques as amyloid sinks which lowered levels of circulating sAβ, thus preventing synaptic impairment early in the disease pathology [77]. These changes of functional plasticity and excitability are not visible at the ultrastructural level without performing complex and extensive synaptic analysis, and are the focus of future investigations.

While our studies separate plaque-associated tissue from apparently healthy tissue, we cannot overlook the probability that soluble amyloid was present. Amyloid plaques are electron-dense, but soluble amyloid is not distinguishable in EM without immunostaining. Three-dimensional EM studies focused on fAβ-neuronal interactions found intimate contacts of amyloid with dendritic membranes in the hippocampus of 3XTg model mice and in the prefrontal cortex of aged canines [78]. It is possible the phenotypic changes we identified in “healthy” hippocampal regions of our transgenic mice are driven by soluble amyloid. This is particularly relevant when interpreting the increased microglial encirclement of dendritic spines in APP^Swe^-PS1Δe9 mice, and microglial interactions with the synaptic neuropil observed in the human AD hippocampus. Additionally, as our human data only focuses on microglial interactions in AD, we cannot overlook the possibility that some of the microglial ultrastructural changes may be driven by normal aging and not solely due to AD pathology.

Our studies have shown that microglial processes in subregions with fAβ plaques contain intracellular fibrillar materials, contrary to microglia among dystrophic only or healthy subregions. This is in line with numerous in vitro and in vivo studies demonstrating microglial capacity to phagocytose both soluble and fibrillar forms of Aβ [79–81]. This finding additionally suggests that microglia may remain in close proximity to amyloid after recognition via cell-surface receptor complexes, as supported by previous two-photon in vivo imaging observations in the cerebral cortex of AD model mice [71]. While microglia are proficient at phagocytosing amyloid, when presented with chronic high levels of fAβ such as measured in AD mouse models and human pathology, their phagolysosomal system becomes overloaded and unable to properly degrade the fAβ [82]. In fact, in vitro studies have found intact fAβ remains within microglia as long as 20 days after incubation with amyloid [83]. This may explain both the high prevalence of fibrillar materials within microglial processes and their increased numbers of lysosomes we reported. Plaque-associated microglial processes particularly had increased numbers of secondary lysosomes, which are often referred to as phagolysosomes—the organelles created when phagosomes and lysosomes fuse to reduce pH and degrade internalized materials [62, 84]. Interestingly, failure to acidify phagolysosomal compartments has been implicated in the fAβ overloading of microglia [85]. This failure may also explain the differences in microglial phagocytosis and different ratios of empty versus non-empty inclusions between cell bodies of seemingly healthy versus dystrophic versus plaque-associated subregions of CA1. Perhaps the increase in non-empty microglial inclusions seen in plaque-associated areas is actually due to an impaired digestion of the phagocytosed amyloid.

It is important to note, however, that because our TEM imaging protocol was conducted on single ultrathin sections, we cannot overlook the possibility that phagocytic vesicles may represent pockets of extracellular material not yet fully engulfed, or interdigitations between microglial processes and surrounding neuropil. 3D-EM reconstructions of microglia previously showed no intracellular amyloid in the cerebral cortex of the APP23 mouse model [86]. However, the differences in mouse model, age of animals, studied region, and sample preparation make it difficult to compare with our own findings. Additionally, this work did not investigate microglial phagocytosis within the synaptic neuropil, and focused on fAβ alone. It is known that microglia readily internalize and degrade numerous targets in healthy and diseased tissue, and their identification should be the focus of future nano-scale resolution studies [87–89]. Other microglial phenotypes or subtypes, such as dark microglia, and others showing intermediate stages of subcellular anomalies (e.g., ER dilation, condensation of cytoplasmic and nucleoplasmic contents), as described in this study, might also behave differently toward amyloid.

Recent transcriptome analysis of human AD tissue lastly revealed similarities and differences in the innate immune response compared to animal models of AD [90]. Nearly all amyloid mouse models of AD yield excessive overexpression of human APP protein, often with multiple mutations driving Aβ_42_ expression to drive plaque formation [91]. These models do not express intracellular tau depositions and often do not recapitulate the extensive neuronal loss documented in human AD cases. It is thus crucial to verify findings in human studies whenever possible. For the first time, we have investigated microglial relationships with the synaptic neuropil at ultrastructural resolution in post-mortem hippocampal tissue derived from individuals who suffered from AD, which allowed us to validate qualitatively our findings with respect to microglial association with extracellular debris and fAβ plaques, encirclement of dystrophic neurites and dendritic spines, as well as phagocytic inclusions.

## Conclusions

Overall, our findings shed more light on microglial ultrastructural diversity in AD amyloid pathology, supporting the previous findings of morphological alterations (enlarged cell bodies), impaired process motility, and phagocytic capacity that were obtained at the light level. This heterogeneity in microglial ultrastructure is paralleled with the diversity recently discovered in single-cell RNAseq studies. In addition, increased microglial process interactions with dendritic spines were uncovered among subregions of AD model mice still unaffected by neuronal dystrophy and plaque pathology, paralleling the functional alterations of neuronal network activity known to be driven by soluble amyloid. Intermediate stages between the typical and dark microglia were further described, raising the intriguing possibility that these dark cells could derive from typical microglia in a stepwise fashion, similarly to the DAM and MGnD recently identified. Future studies are warranted to determine the physiological relevance of these microglial alterations, phenotypic transformations, or subtype expansion, together with their outcome on synaptic loss and neurodegeneration in AD, across different stages of pathology and brain regions.

## Abbreviations

AD: Alzheimer’s disease
Aβ: Amyloid beta
DAB: Diaminobenzidine
DAM: Disease-associated microglia
ER: Endoplasmic reticulum
fAβ: Fibrillar amyloid beta
GWAS: Genome-wide association study
H_2_O_2_: Hydrogen peroxide
IBA1: Ionized calcium binding adaptor molecule 1
IL-1β: Interleukin-1 beta
MGND: Microglia neurodegenerative phenotype
PB: Phosphate buffer
PBS: Phosphate-buffered saline
RT: Room temperature
sAβ: Soluble amyloid beta
SEM: Standard error of the mean
TBS: Tris-buffered saline
TNFα: Tumor necrosis factor alpha
TREM2: Triggering receptor expressed on myeloid cells 2
